# Tobacco Control Policy in Scotland: A Qualitative Study of Expert Views on Successes, Challenges and Future Actions

**DOI:** 10.3390/ijerph16152659

**Published:** 2019-07-25

**Authors:** Yvonne Laird, Fiona Myers, Garth Reid, John McAteer

**Affiliations:** 1 Scottish Collaboration for Public Health Research and Policy, University of Edinburgh, Edinburgh EH1 2QL, UK; 2 Evidence for Action, NHS Health Scotland, Edinburgh EH12 9EB, UK

**Keywords:** tobacco smoking, cigarette smoking, legislation, cessation, thematic analysis, qualitative research

## Abstract

The Scottish Government launched a tobacco control strategy in 2013 with the ambition of making Scotland tobacco smoke-free by 2034. However, 17% of the adult population in Scotland smoke cigarettes. This study aimed to provide insight into why policies are successful or not and provide suggestions for future policy actions. Individual interviews with ten tobacco control experts were conducted and the results were analyzed using thematic analysis. Key successes included strong political leadership, mass media campaigns, legislation to address availability and marketing of cigarettes and tobacco products, and legislation to reduce second-hand smoke exposure. Challenges included implementing policy actions, monitoring and evaluation of tobacco control actions, addressing health inequalities in smoking prevalence, and external factors that influenced the success of policy actions. Key suggestions put forward for future policy actions included addressing the price and availability of tobacco products, maintaining strong political leadership on tobacco control, building on the success of the ‘Take it Right Outside’ mass media campaign with further mass media campaigns to tackle other aspects of tobacco control, and developing and testing methods of addressing inequalities in cigarette smoking prevalence. The findings of this study can inform future tobacco control policy in Scotland and have relevance for tobacco control policies in other countries.

## 1. Introduction

The well-established harmful effects of cigarette smoking have led to global action to reduce tobacco smoking prevalence [1], including the introduction of a number of tobacco control policies by governments across the world. In 2013, the Scottish Government launched a national tobacco control strategy with the aim of making Scotland tobacco smoke-free by 2034 [2]. The strategy outlined 46 proposed actions broadly focused on cigarette smoking cessation services and initiatives, prevention of smoking uptake, reducing exposure to second-hand smoke, and reducing exposure to tobacco marketing. 

Between 2013 and 2016, tobacco smoking prevalence remained relatively static, with an approximate prevalence of 21% of the adult population in Scotland who smoked cigarettes. The most recent prevalence data suggests tobacco smoking has declined, with 17% of the adult population in Scotland who smoke cigarettes [3]. Prevalence of tobacco smoking is particularly high for those in the most deprived areas, where 30% of adults smoke compared with 10% in the least deprived communities in Scotland. In order to achieve the Scottish Government’s ambition of making Scotland tobacco smoke-free by 2034, and to address the economic and health burdens associated with smoking cigarettes [4,5], it is essential to understand the effectiveness of policy actions to reduce tobacco consumption. A review of the 2013 tobacco control strategy evaluated the impact of policy actions to reduce tobacco consumption, and highlighted promise for policies aimed at reducing children’s exposure to second-hand smoke, the implementation of a tobacco display ban in retail outlets, a mass media campaign ‘Take it Right Outside’, smoke-free hospital grounds, and the introduction of plain packaging legislation [6]. This review complements evaluations of tobacco control actions in Scotland and the UK which have identified promising effects (as well as challenges) for policies tackling second-hand smoke exposure [7,8], the implementation of a peer-led smoking prevention intervention in schools [9,10], mass media campaigns [11], tobacco packaging legislation [12], and point-of-sale legislation [13,14]. Globally, systematic review evidence has identified promising effects for tobacco control policies and interventions on reducing cigarette smoking prevalence and reducing inequalities in cigarette smoking [15,16,17,18,19], and observational research provides evidence for the implementation of multiple policy actions to reduce smoking prevalence [20]. A review of European tobacco control actions found that tobacco control efforts are often hampered by tobacco industry interference, with the authors outlining a need for strong political leadership to tackle cigarette smoking [21]. These studies highlight the potential promise for a range of tobacco control policy actions, however given the prevalence of cigarette smoking in Scotland, particularly in the most deprived communities, further progress to reduce cigarette smoking is needed. Exploring expert perspectives on the success of policy actions could provide insight into why policies are successful or not and inform the development of future policy actions to tackle tobacco consumption.

The aims of this study were: (1) to identify successes and challenges of the policy actions outlined in the 2013 tobacco control strategy for Scotland as perceived by experts in tobacco control; and (2) to identify recommended actions for the 2018 tobacco control strategy for Scotland [22]. A summary of the findings of this research was previously published to inform the 2018 tobacco control strategy for Scotland [23].

## 2. Materials and Methods 

Individual semi-structured interviews were conducted with experts in the tobacco control field in Scotland. The six-step process of thematic analysis outlined by Braun and Clarke was followed [24], as thematic analysis provides a flexible and reflective approach to obtain a rich understanding of experts’ experiences and perspectives of tobacco control policy in Scotland. Ethical approval was obtained from the Usher Research Ethics Group at the University of Edinburgh (Ethical approval code 1713).

### 2.1. Participants

Participants included tobacco control experts. Experts were identified through consultation with the chair of the Research and Evaluation Subgroup for Tobacco Control within the Scottish Government, National Health Service (NHS) Health Scotland, and government officials. Individuals were invited to participate if they: (1) were a policy maker, practitioner or researcher; (2) were considered a leader in tobacco control in Scotland (e.g., professor, senior public health practitioner, senior civil servant); and (3) had extensive experience in one or more areas of tobacco control in Scotland. Care was made to ensure the final group of participants represented a comprehensive coverage of tobacco control policy areas in Scotland, and included representatives across government, frontline NHS and other practitioners, and senior researchers. A total of 12 experts were invited to participate and 10 agreed to take part in an individual interview.

### 2.2. Procedure

Each participant participated in one face-to-face or telephone interview with a member of the research team (Y.L. or F.M.). Interviews were audio-recorded and later transcribed and lasted an average of 54 minutes (range 30–79 minutes).

### 2.3. Instrument

A semi-structured interview guide was developed to identify perceived successes and challenges of tobacco control policy actions, and recommendations for the 2018 tobacco control strategy. Questions were designed to elicit open responses and contained accompanying prompts and probes. The interview guide was structured around five broad areas relating to the actions outlined in the 2013 strategy, including tobacco prevention, tobacco cessation, smoke-free environments, marketing, and illicit tobacco control actions. The interview guide was piloted prior to use, and some of the questions were modified for clarity following this. 

### 2.4. Analysis

Thematic analysis was undertaken following the six step process outlined by Braun and Clarke [24]. Two members of the project team (Y.L. and J.M.) independently read and coded two of the transcripts. Following this, an initial thematic structure for analysis was agreed between the team members. Transcripts were then uploaded to NVivo10 software (QSR International Pty Ltd, vs. 10, 2012, Melbourne, Australia), after which one of the researchers analyzed the remainder of the transcripts (Y.L.). This involved the identification and coding of themes and sub-themes. These were continually reviewed until a final set of themes were constructed. Another researcher (J.M.) reviewed the final set of themes to check that these were representative of the data. 

## 3. Results

Findings are organized under three main themes: (1) perceived successes; (2) perceived challenges; and (3) future policy actions. 

### 3.1. Perceived Successes

Six sub-themes were identified in relation to participants’ perceptions of successes, including: political leadership, mass media, availability, cessation, smoke-free environments, and prevention (see Table 1). 

Political leadership was deemed by many of the participants to have contributed to the success of the strategy. Participants felt that the existence of the strategy demonstrated the government’s commitment to tobacco control and gave clear action points and targets to work towards. Several participants felt that the bold targets and policies introduced by the Scottish Government, such as being the first nation to introduce a target for a smoke-free generation, have led to strong international exposure and have placed Scotland as a leader internationally for tobacco control. Participants felt that the establishment of a Ministerial Working Group on Tobacco Control, with ‘Prevention’ and ‘Research and Evaluation’ sub groups, helped to focus efforts and drive action. Strong political leadership helped to establish collaborations between multiple sectors, helped to ensure youth involvement in developing solutions, and demonstrated the Scottish Government’s commitment to tobacco control. Linked to this, participants spoke positively about the quality of tobacco control research in Scotland and the strong links between research, government and policy.

“I do think the government have done really well in this area, over the last, if we go back to 2005 onwards. It’s been an area, I guess, that we’ve led on internationally. And also, I would say that it has led to a marvelous academic research base here, that is much larger than it would be otherwise… I think that is as a result of the lead out of the government on this area.”

Specific initiatives were highlighted as examples of success. There was consensus amongst participants about the success of the ‘Take it Right Outside’ mass media campaign (http://www.rightoutside.org), which highlighted the dangers of second-hand smoke exposure. Participants suggested that the campaign contributed to a culture shift in Scotland in relation to exposure to second-hand smoke, with members of the public perceiving it to be less acceptable to expose others to second-hand smoke. Participants also emphasized the success of policy actions tackling the availability of tobacco products. Increasing the age for purchasing tobacco products (to >18 years old), introducing plain packaging legislation, minimum pack sizes and the display ban were considered particularly successful. It was felt that these policy actions could reduce susceptibility to smoking cigarettes amongst young people in particular. Participants also highlighted progress made towards the control of illicit tobacco. With regards to tackling cessation of tobacco, the focus on inequalities, a review of cessation services, introduction of specialist smoking cessation services, carbon monoxide monitoring and opt-out referrals for pregnant women, and progress towards establishing national branding for smoking cessation services were considered successful actions.

There were mixed views on the success of legislation relating to smoke-free environments. Most participants felt that legislation to ban smoking in cars with children was a positive step. However, the success of legislation for smoke-free hospital grounds was less clear. Some participants felt that the introduction of this legislation posed a challenge in terms of enforcement (see perceived challenges), whilst others felt that the legislation on smoke-free hospital grounds has and will continue to contribute to a shift in culture in relation to the perceived acceptability of exposing others to second-hand smoke. Finally, introducing ‘A Stop Smoking in Schools Trial’ (ASSIST) in several schools in Scotland was identified as a successful tobacco prevention action, but also a challenge by some participants due to the current low levels of smoking in Scottish youth (see perceived challenges). 

Several participants felt that the successes outlined above, and the combined effect of numerous policies and actions on tobacco control in Scotland, have led to a marked change in national culture in relation to reduced public acceptability of smoking cigarettes. It is also important to note that participants felt that any potentially beneficial effects of some policy actions, in terms of smoking prevalence, might not become apparent for several years. For example, it was suggested that plain packaging legislation might not have an immediate impact on susceptibility to smoking in young people but instead contribute to a longer-term shift. 

### 3.2. Perceived Challenges

Four sub-themes were identified in relation to participants’ perceptions of challenges, including: (1) implementation; (2) monitoring and evaluation; (3) health inequalities; and (4) external factors (see Table 1). 

#### 3.2.1. Implementation

Specific challenges were identified in relation to three initiatives: smoke-free hospital grounds, pharmacy cessation services, and the ASSIST prevention programme. 

Participants felt that the legislation on smoke-free hospital grounds was a positive step, but that it would take time for a culture shift to occur. One participant expressed concern that the public may not understand the rationale behind the policy, and others felt that it was potentially stigmatizing, particularly for vulnerable people who may have difficulty walking to hospital perimeters to smoke. Some participants suggested that smoking shelters might be a more appropriate solution. 

Participants also felt that pharmacy cessation services could free up resources to focus on other areas of tobacco control but felt that efforts were needed to first make pharmacy services the best they could be. Participants felt that the quality of such services was variable, and that this needed to be addressed. The ASSIST programme was also identified as a challenge, both in terms of the financial resources required for national roll-out of the programme, and decisions related to where and when to implement the intervention. For example, participants noted that earlier intervention of the programme may be beneficial in some areas of disadvantage where smoking uptake may occur at an earlier age. There were several suggestions made by participants that resources were not being used as effectively as they could be, with one participant suggesting that rolling out scalable interventions is something that needs to be improved upon. 

Lack of resources was consistently identified as a challenge when implementing policy actions. Several participants suggested that services need further investment and that due to limited funding, difficult decisions had to be made about where to focus resources. Workforce development was also identified as a challenge, particularly when staff change roles leaving no one to drive forward particular actions. In addition, participants felt that there needed to be designated staff, as without staff to oversee actions, they are less likely to be implemented. Changing structures of NHS delivery also presented a challenge for implementation. 

Sustaining momentum was discussed by many of the participants as a challenge. Participants expressed frustration over slow progress on smoke-free prisons. Participants also expressed frustration over recommendations or findings not being implemented, for example pharmacies not prescribing recommended cessation drugs. 

#### 3.2.2. Monitoring and Evaluation 

Challenges were expressed in relation to NHS tobacco control performance targets being too broad (in relation to deprivation) and being unachievable for some (cessation targets). It was also felt that one-year targets did not give practitioners enough time to plan and implement actions. 

Many participants identified challenges in relation to measuring and demonstrating impact, particularly where an immediate impact or change from policy actions would not be expected. For example, the display ban or plain packaging legislation would not necessarily be expected to have an immediate impact on susceptibility to smoking in young people. Participants also highlighted that it can be challenging to tease out what has worked, and that the proper evaluation of the impact of policy actions would require substantial resource. 

#### 3.2.3. Health Inequalities

Participants felt that the focus on inequalities in smoking in the strategy was a strength, but that the difficulty of reaching the most deprived was underestimated, with the majority of participants highlighting this as a challenge. Participants expressed concerns that some tobacco control actions could stigmatise the most disadvantaged, for example the ‘Take it Right Outside’ campaign could stigmatise some mothers living in areas of high deprivation since it may not always be possible or they may not feel safe to smoke outside of the home. Some participants also felt that the intensity required to intervene in the most disadvantaged areas was lacking, and echoing this, others commented that more targeted approaches are needed to reach people in these areas.

“I think we need to recognise smoking as part of a much broader public health and social issue… it is actually part of people’s lives as a social practice, and I think we recognise the place it has in people’s lives. We need to be really alert that smoking is a major inequalities issue. And it is not just a driver of inequalities, it is a product of those inequalities. It is heavily related to greater stigmatisation of communities and people, and I think we need to make sure that whatever measures we put in place, we’re not further stigmatising a product or a group that are already highly socio-stigmatised. So, that’s become a challenge, coming back to your earlier question, for tobacco control.”

#### 3.2.4. External Factors

It was suggested that the tobacco industry has attempted to circumvent plain packaging legislation, for example through developing products to store cigarettes that contains advertising. Participants also raised concerns that the tobacco industry has attempted to influence health policy and has sponsored arts and music venues under the guise of social responsibility in England. It was also suggested that some tobacco sales representatives have incentivised small retailers to promote certain brands. It was felt there was a need to monitor the activity of the tobacco industry as well as innovations such as heated tobacco products, and to consider additional legislation.

Proxy sales and illicit tobacco were identified as another ongoing challenge, particularly in the most deprived communities. Incidents of proxy sales were described including children under 18 years being sold tobacco products by some retailers or being bought tobacco products by adults. In relation to illicit tobacco, while some participants felt that the tobacco industry implies that illicit tobacco is a bigger problem than it is, others felt that more enforcement of actions to control illicit tobacco were needed, particularly now that plain packaging legislation may have made illicit tobacco more prevalent. There were concerns raised in relation to the language that was being used in campaigns to prevent the uptake of illicit tobacco. Some participants, for example, felt that describing illicit tobacco as ‘dangerous’ was problematic, given that all tobacco products are dangerous. 

Finally, social media marketing and inadvertent advertising of tobacco products in films, TV and music videos was identified as an ongoing challenge that could potentially hamper the efforts of policy actions. 

### 3.3. Future Policy Actions 

Future policy actions in relation to political leadership, mass media, price and availability of tobacco products, electronic cigarettes (e-cigarettes), smoke-free environments, cigarette smoking cessation, and actions that cut across multiple areas of tobacco control were proposed. These are presented in Table 2. 

Building on what many participants deemed to be a success of the 2013 strategy, participants felt it was important that there remained strong political leadership in relation to tobacco control. Participants felt that this leadership would be necessary to keep tobacco control high on the political agenda and to sustain action and momentum on tobacco control in Scotland. Participants felt that government should continue to invest in existing and new tobacco control services, with protected government budgets for tobacco control work in line with inflation. 

Many participants felt that further mass media campaigns, building on the success of the ‘Take it Right Outside’ campaign, should be developed to focus on other aspects of tobacco control.

The majority of the participants highlighted a need to address the price and availability of tobacco products. Participants put forward a number of suggestions as to how this could be achieved, including introducing minimum pricing on tobacco products, introducing a higher taxation/levy on the tobacco industry, establishing a licencing system/fee for retailers who wish to sell tobacco, less prominent positioning of tobacco products in retail outlets, establishment of a “health cordon” around schools prohibiting the sales of tobacco products, and further raise the age for purchasing tobacco products to 21 years.

“…the big issue I think, and having been involved in various workshops and discussions, thinking about the next steps in terms of tobacco control in Scotland… is the supply and availability of tobacco. There’s 11,000 outlets selling tobacco across Scotland, we have availability which is far in excess of any other data that’s comparable in any other countries, per population, and it widely remains available on every street corner. I think that we’re never going to meet the challenges of a tobacco-free Scotland, without addressing the supply and the availability. So, that would be number one for me, in terms of what’s next”.

The majority of participants felt there was a need to address e-cigarettes in future policy as a harm-reduction tool, in a way that does not undermine other work in tobacco control. A few participants were concerned that e-cigarettes could act as a gateway to cigarette smoking or increase susceptibility to cigarette smoking, particularly amongst young people, although one participant stated that there was no evidence of this and others suggested it was too early to tell if this might be happening. A few people expressed concern that the long-term health impacts of e-cigarettes were uncertain. It was also suggested that the growing popularity and use of e-cigarettes could serve to *“perpetuate the sale and consumption of conventional tobacco products and rehabilitate the manufacturers of those products”.*

Further action to reduce second-hand smoke exposure, through introducing policies around smoke-free environments and monitoring second-hand smoke exposure, was wanted. Specific suggestions included the introduction of legislation on smoke-free parks, playgrounds, school grounds, universities, mental health facilities, and flat stairwells, as well as continued action and progress towards smoke-free prisons. One person also suggested considering further action to reduce second-hand smoke exposure in homes. 

Several participants highlighted the need to develop policies around smoke-free environments in collaboration with the people who use these facilities. One participant expressed concerns about tobacco policies targeting private places, such as homes, and felt that the success of previous tobacco control policies was that they enhanced liberty, choice and freedoms of non-smokers. Comparatively, the same participant felt that measures around smoke-free private places could stigmatise the most deprived smokers. Indeed, others also mentioned that an unintended consequence of introducing legislation around smoke-free parks or other public spaces might be to further stigmatise the most deprived groups. Participants also made suggestions in relation to monitoring and targets, including routine salivary cotinine tests for children to capture objective data on second-hand smoke exposure, and establishing a target for second-hand smoke exposure. One participant put forward a specific suggestion for a second-hand smoke exposure target of 3 out of 4 adults with no evidence of exposure to second-hand smoke by 2020. 

Additional policy actions in relation to tobacco cessation were proposed. In particular, participants wanted to see a broader range of NHS practitioners equipped to deliver advice on smoking cessation, a single brand to align all smoking cessation services across NHS boards, improved training for smoking cessation staff, establishing a new local delivery plan standard for cessation services with a focus on inequalities, and improving the quality and availability of pharmacy cessation provision. 

Further suggestions cut across multiple areas of tobacco control. This included having a focus on particular groups including 16–24-year-olds, people with mental health conditions, looked after and accommodated young people, and pregnant women. One participant suggested joining up tobacco control initiatives with dentists and General Practitioners (GPs), and considering other ways of engaging people. Another suggestion was the introduction of tobacco and smoking awareness training to the undergraduate curriculum for all health professionals. There was consensus amongst the participants that future actions should address inequalities in smoking, with some participants suggesting a need to focus interventions in the most deprived areas. There was a suggestion to transfer resources between health boards to more deprived areas, with a recognition that this could be a challenging conversation. Participants also suggested a need to develop an evaluation strategy with robust monitoring and measurable actions. One participant suggested that routine monitoring could use more sophisticated techniques such as econometrics, and another felt that more detail in routine monitoring data could be helpful (e.g., to gain an understanding of where declines in uptake of cessation services are occurring). Several participants also felt that future actions should be non-intrusive and reduce stigma. Finally, participants suggested a need for future policy actions to connect with other policy priority areas and health behaviours, such as policies related to obesity and alcohol use or connect to policies around children and families. Linked to this, some participants felt there should be more collaborative working with organisations that target other health behaviours or social priorities. 

## 4. Discussion

Experts in tobacco control in Scotland provided their views on the strengths and challenges of the 2013 tobacco control strategy in Scotland and put forward recommendations for future policy actions. Strong political leadership, mass media campaigns, and legislation to reduce second-hand smoke exposure and address availability and marketing of tobacco products were identified successes from the 2013 strategy. Implementation of policy actions, monitoring and evaluation, and addressing health inequalities were identified as key challenges. The challenges identified may, to some extent, explain why smoking prevalence remained relatively static in Scotland for the first four years following the implementation of the 2013 tobacco control strategy. The participants outlined how some policy actions took longer than anticipated to implement, that enforcement of policies was difficult and the challenge of reaching the most deprived communities where smoking rates are highest. In addition to the identified challenges, policy actions may not have had an immediate effect on smoking prevalence. Data suggests that some policy actions have positively influenced outcomes which may contribute to a shift in smoking prevalence, for example by positively changing perceived attitudes towards smoking, susceptibility to smoking and perceived accessibility of smoking [18,25]. The longer than intended implementation of policy actions and the time for change to occur in smoking prevalence may explain why change in smoking prevalence was not observed until 2017, four years after the publication of the strategy. Additionally, the number of non-smokers exposed to second-hand smoke has decreased [8]. Further monitoring and evaluation is necessary to understand longer-term impacts of policy actions, and whether observed changes in prevalence of cigarette smoking is sustained. 

Experts expressed a need to build on the successes of the previous strategy and put forward a number of suggestions for future policy actions, with participants identifying a particular need to tackle the price and availability of tobacco products. Existing research suggests that the availability of tobacco outlets is associated with starting or continuing to smoke [26], and that outlets typically cluster in areas of deprivation [27]. Whilst existing intervention evidence on tobacco outlet density is lacking, modelling studies provide preliminary evidence for the potential for policies aimed at reducing the availability of tobacco retail outlets on smoking prevalence [28]. Raising the price of tobacco products also has promise to reduce smoking prevalence, particularly amongst disadvantaged socioeconomic groups [29,30,31,32]. As such, these policies may have potential, crucially, to reduce inequalities in smoking in addition to other tobacco control policies and interventions. Tobacco harm-reduction tools, predominantly e-cigarettes, were also put forward by several of the participants as an action that should be addressed in the next tobacco control strategy. Whilst some participants had concerns about the safety and use of e-cigarettes and their connection to the tobacco industry, most of the participants felt that e-cigarettes represented a potential harm-reduction tool that could help people to quit smoking cigarettes. E-cigarettes have been estimated to be up to 95% less harmful than cigarette smoking [33], and emerging evidence suggests that e-cigarettes may help people to quit cigarette smoking, although more high-quality research is needed to better understand their effectiveness and their safety [34]. 

This study adds to previous tobacco control policy research [20,35,36,37] by providing a rich qualitative account of expert views of the successes and challenges of tobacco control policy in Scotland, covering a breadth of tobacco policy areas. Participants were leaders in their respective areas and included policy makers, practitioners, and researchers. Participants were well positioned to give a comprehensive insight into tobacco control policy in Scotland in their respective areas of expertise, which is a strength of the current study. This work was conducted alongside the development of tobacco control policy in Scotland, with a previously published summary of the findings [23] directly informing the 2018 tobacco control strategy in Scotland [22]. As such, we provide an example of how research can run alongside policy development to facilitate the development of evidence informed public health policy. There are also limitations to the study. Due to the nature of the study, participants may have placed greater emphasis on their own areas of expertise when asked for suggestions in relation to future policy actions and the findings are reliant on a relatively small group of experts who are likely to be part of similar professional circles. As such, there is possible bias in the findings due to the nature of the sample. However, suggestions provided detailed accounts of multiple tobacco policy areas, and participants generally provided evidence to support their positions. A further limitation to the study was that only experts were included. Including perspectives of the public, the tobacco industry and tobacco retailers may have provided insight into the acceptability of proposed tobacco control policy and future actions, for example obtaining perceptions on the feasibility and acceptability of the popular suggestion by experts to tackle the price and availability of tobacco products. 

These findings further highlight the importance of strong political leadership to drive forward tobacco control policy [21,38], which participants attributed as key to implementing actions and policies that participants deemed to be successful, including mass media campaigns, policies to reduce availability of tobacco products and policies relating to smoke-free environments. Reflecting on the successes and challenges of the 2013 tobacco control strategy provides an opportunity to build on and address these in future policy actions, and enabled experts in Scotland to direct these future policy actions. 

The generalisability of findings to other countries and settings is unclear, as different cultural norms, cigarette smoking prevalence rates, and other factors could influence the success of policy actions to address smoking prevalence. As such, further research reflecting on and evaluating the successes and challenges of multiple policy actions within different countries and contexts would add to the findings of the current study, in addition to natural experiments and controlled trials to evaluate policy actions. 

## 5. Conclusions

This study provides a rich qualitative account of tobacco control policy in Scotland between 2013 and 2018, experts’ views on the successes and challenges of these policy actions, and suggestions for future policy actions. Participants felt that future policy actions should build on the successes of the 2013 strategy, and, in particular, tackle the price and availability of tobacco products, develop further mass media campaigns, and address challenges in relation to implementation, monitoring and evaluation, and health inequalities in smoking prevalence. Additionally, the majority of participants felt that e-cigarettes should be addressed in the next tobacco control strategy as a potential harm-reduction tool. This study adds to previous research by providing a qualitative evaluation of tobacco control policy, which can be used in conjunction with quantitative policy evaluation to provide insight into why tobacco control policies are successful or not. This study also provides an example of how research can run alongside public health policy development. 

## Figures and Tables

**Table 1:** Perceived successes and challenges of the Tobacco Control Strategy for Scotland.

Theme	Sub-Theme	Codes
**Perceived Successes**	Political leadership	Existence of strategy itself Bold targets and policies Ministerial Working Group with sub-groups on (1) Prevention and (2) Research and Evaluation Collaborations between multiple sectors Youth involvement in developing solutions Commitment to research and evaluation
	Mass media	‘Take it Right Outside’ campaign
	Availability	Increasing age for purchasing tobacco products to 18 Policies relating to tobacco marketing (e.g., display ban, plain packaging, minimum pack size) Enforcement of illicit tobacco
	Cessation	Focused efforts on inequalities Review of cessation services Specialist cigarette smoking cessation services Carbon monoxide monitoring and opt-out referrals for pregnant women Progress towards establishing national branding for cessation services
	Smoke-free environments	Legislation banning smoking in cars with children Smoke-free hospital grounds [*however, implementation identified as a challenge*]
	Prevention	‘A Stop Smoking in Schools Trial’ (ASSIST) [*however, implementation identified as a challenge*]
**Perceived Challenges**	Implementation	Public may not understand rationale for policy actions Less stigmatising solutions needed Variable quality of cessation services Lack of resources Difficulty making decisions on where to implement interventions Workforce development/lack of staff to drive actions forward Changing organisational structures Sustaining momentum
	Monitoring and evaluation	National Health Service performance targets too broad and/or unachievable Measuring and demonstrating impact
	Health inequalities	Difficulty reaching most deprived Some tobacco control policies could stigmatise the most disadvantaged Intensity required to intervene in the most deprived areas lacking
	External factors	Tobacco industry Proxy sales and illicit tobacco Advertising of tobacco products via social media, film, TV and music videos

**Table 2:** Summary of proposed future Tobacco Control policy actions for Scotland (as proposed by study participants).

Policy Area	Future Proposed Action
**Political Leadership**	Strong political leadership Sustained action and momentum Continued investment in existing and new services relevant to tobacco control Protect government budgets for tobacco work in line with inflation
**Mass Media**	Mass media campaigns to tackle other aspects of tobacco control (following on from the success of the ‘Take it Right Outside’ campaign)
**Price and Availability**	Introduce a minimum pricing structure Introduce a taxation or levy on the tobacco industry Establish a licencing fee for tobacco retailers Less prominent positioning of tobacco in retail outlets Establishment of a “health-cordon” around schools prohibiting sales of tobacco products Further raise the age for purchasing tobacco products to 21
**E-cigarettes**	Address e-cigarettes in the new strategy as a harm-reduction tool in a way that does not undermine other work in tobacco control
**Smoke-Free Environments**	Smoke-free parks, playgrounds, school grounds, universities, mental health facilities and flat stairwells Consider further action to reduce second-hand smoke exposure in homes Continue actions and progress related to smoke-free prisons Develop policies in collaboration with the people who use those facilities Routine salivary cotinine tests for children to capture objective data on second-hand smoke exposure Establish a target for second-hand smoke exposure. Specific suggestion put forward: 3 out of 4 adults with no evidence of exposure to second-hand smoke by 2020
**Cessation**	Equip a broader range of NHS staff to deliver advice on smoking cessation A single brand to align all smoking cessation services across health boards Better training for smoking cessation staff Establish a new local delivery plan standard for cessation services with a focus on inequalities Improve the quality and availability of pharmacy cessation provision
**Cuts across Multiple Areas**	Focus on specific populations, e.g., 16–24 year-olds, people with mental health conditions, looked after and accommodated young people, and pregnant women Consider new ways of engaging people, e.g., joining up stop smoking services with dentists Introduction of tobacco and smoking awareness training to the undergraduate curriculum for all health professionals Focus interventions in the most deprived areas Development of an evaluation strategy, robust monitoring and measurable actions Future actions should be non-intrusive and reduce stigma Connect tobacco with other policy priorities, e.g., obesity, alcohol use, non-communicable diseases More collaborative working with organisations that target other health or social care priorities (e.g., mental health charities, youth organisations)
